# Sleep disturbance and psychotic-like experiences among urban adolescents with and without parental migration

**DOI:** 10.3389/fpubh.2022.1037963

**Published:** 2023-01-04

**Authors:** Dongfang Wang, Zijuan Ma, Shuyi Zhai, Meng Sun, Fang Fan

**Affiliations:** ^1^Guangdong Key Laboratory of Mental Health and Cognitive Science, Ministry of Education Key Laboratory of Brain Cognition and Educational Science, Centre for Studies of Psychological Applications, School of Psychology, South China Normal University, Guangzhou, China; ^2^Faculty of Medicine, McGill University, Montreal, QC, Canada; ^3^Department of Social Psychiatry, The Affiliated Brain Hospital of Guangzhou Medical University (Guangzhou Huiai Hospital), Guangzhou, China

**Keywords:** sleep disturbance, psychotic-like experiences, parental migration, school climate, family function

## Abstract

**Aim:**

Sleep disturbance was closely associated with an increased risk of psychotic-like experiences (PLEs). This study aims to explore the association between sleep disturbance and PLEs among urban adolescents with and without parental migration.

**Methods:**

A total of 67, 532 urban Chinese adolescents were recruited in a large web-based survey during April 21st to May 12th, 2021. In our study, sleep disturbance, PLEs, family function, school climate, and a series of socio-demographic were assessed. And hierarchical logistic regression analyses were performed to examine influential factors associated with PLEs.

**Results:**

Urban left-behind children (LBC) had a higher prevalence of sleep disturbance and PLEs than non-LBC. After controlling for confounders, parental migration was associated to PLEs with weak significance (OR = 1.19). Meanwhile, sleep disturbance was found to be a robust risk factor for PLEs (OR = 3.84 and 4.09), with or without the effect of parental migration. In addition, better family function and school climate has significant association with decreased risk of PLEs.

**Conclusion:**

Adolescents with sleep disturbance are more likely to report PLEs. Adolescents' PLEs preventive strategies could focus on reducing sleep disturbance related symptoms as well as improving family function and school climate.

## Introduction

Psychotic-like experiences (PLEs) refer to the occurrence of positive symptoms that resemble those of psychosis in the absence of a full-blown psychotic disorder (1). PLEs are not uncommon (2), especially in adolescents, with 17% of children aged 9–12 and 7.5% of adolescents aged 13–18 years having PLEs (3). Among the Chinese population, 49.3% adolescents reported having at least one PLE within 1 month, while 15.4% experienced high frequent PLEs (4). Theoretical models and practical research both revealed that PLEs in early age were associated with an increased risk of subsequent psychotic disorders (5, 6) and non-psychotic disorders (7, 8). Therefore, exploring the risk factors of PLEs may provide useful information in understanding the development of mental disorders and throw light on the prevention of adolescents' mental health problems.

Sleep disturbance, such as insomnia and poor sleep quality, can be involved in the initiation and development of psychological problems among adolescents (9, 10). Sufficient research also shows that sleep disturbance is one factor that appears to elevate the risk for the occurrence of PLEs. Theoretically, sleep disturbance may impair stress tolerance, immune function, and cognitive function, and exacerbate social-emotional distress (11, 12), all of which are thought to play critical roles in the etiology of psychosis (13, 14). Recently, our team confirmed the relationship between sleep disturbance and PLEs in an adolescent group based on a large sample of survey, concluding that sleep disturbance was significantly associated with increased risk for PLEs, as well as that sleep disturbance predicted the new onset and persistence of PLEs (15, 16). Collectively, sleep disturbance may serve as an important indicator for early screening and intervention for PLEs among adolescents. Whether the role of sleep disturbance is stable for different specific groups of adolescents, however, remains unclear.

Existing research supports that parental migration is detrimental to the health of children and adolescents (17). Since the Chinese government implemented the “*Reform and Opening up*” policy in 1978, millions of Chinese labors has migrated from rural to urban areas in search of better-paying employments and life opportunities. One consequence of this migration is that most migrants have to leave their children in their home communities, and a large population of left-behind children (LBC) was yielded. Given that there are over 68 million rural LBC according to the governmental data (18), parental migration is a significant public health concern in China. According to attachment theory, secure parent-child attachments are the foundation for the stability and security children need to grow and develop (19, 20). Parents leaving their children when they particularly need a stable environment can lead to adverse consequences for LBC, including subjective feelings of abandonment and confusion about who to turn to for parental support; feelings of emotional vulnerability; and exposure to violence from primary and proxy caregivers (21).

A growing number of studies have found LBC suffer from more mental health problems compared to their non-left behind peers, such as depressive symptoms (22), anxiety (23), and suicidality (24). However, limited research has examined the association between parental migration and PLEs. Within such few studies, the temporary relationship between parental migration and PLEs appears inconclusive. In the study by Sun et al. (25), they found that among Chinese adolescents from rural Xiangxi region and Changsha city of Hunan province, LBC reported higher scores in PLEs evaluation than non-LBC, despite no significant differences in the prevalence of frequent PLEs. In their subsequent study, Sun et al. (26) reported that LBC among 5, 427 adolescents aged between 10 and 16 were not at a higher risk for PLEs, while those aged 10 to 23.3 were likely to have frequent PLEs in a sample of 8, 729 adolescents (27). Therefore, it would be worthwhile to examine the impact of parental migration on adolescent PLEs by examining a larger sample in other regions. Furthermore, China has experienced rapid urbanization in recent decades. As a consequence of this drastic growth in urban population, urban vs. rural population ratio has been increased (28). Since 2015, rural-to-urban labors migration in China has started to decline, while urban-to-urban migration has henceforth been increasing, leading to a large and ever-growing population of urban LBC (29). From 2005 to 2015, it is estimated that the number of urban LBC increased from 14.7 to 28.3 million (30). Limited study also demonstrated that urban LBC experienced more mental health problems than rural LBC (31). Despite growing evidence on the association between parental migration and rural LBC's mental health, there is a lack of literature addressing this association for urban LBC, particularly in the context of urban-to-urban migration in China.

Furthermore, LBC often have less parental supervision, which increases their sleep disturbance. Indeed, Tang et al. (32), in 3,346 Chinese adolescents, found that LBC have higher risk of sleep disturbance than the children with non-migrant parents (32). Previous work also identified LBC as a vulnerable population who may be particularly affected by sleep disturbance (33). Therefore, we suspect that parental migration may exacerbate the role of sleep disturbance on adolescents with PLEs. Accordingly, we conducted a cross-sectional study based on a large sample of urban high school students from a Southern province in China to examine the relationship between sleep disturbance, parental migration, and PLEs. In the survey, several important confounding factors need to be considered. In addition to the socio-demographic characteristics [e.g., sex (34), age (27)] that has been shown to be potentially related to PLEs, the environmental factors (family and school) in which the adolescents were exposed to need to be examined. The current study used family function and school climate to represent two environmental factors of family and school respectively. Family function reflects the structural and organizational properties of a family group as well as the patterns of interactions between family members, which include diverse dimensions, such as family communication, affective expression and parental involvement (35). School climate refers to the relatively stable property of school environment that is experienced by its members (36). In our prior study, family function and school climate have been found to serve as protective factors against PLEs (4).

From previous research, therefore, it was hypothesized that:

Hypothesis 1: The prevalence of sleep disturbance and PLEs in LBC is significantly higher than that in non-LBC.

Hypothesis 2: Sleep disturbance have a negative effect on PLEs after controlling for demographic characteristics, family function and school climate.

Hypothesis 3: The association between sleep disturbance and PLEs might differ between LBC and non-LBC.

## Methods

### Procedure

During April 21st to May 12th, 2021, we conducted a cross-sectional web-based survey in 117 schools in one district of Shenzhen in Guangdong province. Shenzhen is the first special economic zone in China, and its economic level is at the forefront of the country, which is also one of the cities with the largest labor migration between cities in China. According to statistics, the resident population in Shenzhen at the end of 2021 and the beginning of 2022 is ~17.7 million, of which 68.5% are resident non-local household registration population (37). Meanwhile, data of the local education bureau estimated that the number of junior or senior high school students in the district was ~1,26,000. The survey was supported by the local school board and was completed with the assistance of 6 research assistants and the supervising teacher of each school. Before the online survey, the local education bureau and schools jointly sent out electronic survey invitation letter to students and their guardians. After obtaining the electronic informed consent from students and their guardians, the researcher would send out the Quick Response (QR) code or send the link of the questionnaire to participants. Students were asked to scan the QR code or click the link through mobile phones or computers to complete questionnaires. Responses to the survey were kept confidential and all participants could withdraw at any time during the test. The details of the study design and sample procedures have also been described elsewhere (4).

The study was carried out in accordance with the Helsinki Declaration as revised in 1989 and approved by the Ethics Committees of South China Normal University (SCNU-PSY-2021-094). Trainings (e.g., students' psychological crisis intervention, counseling skills) were provided to all teachers through online or off-line classes in case any of the participants required professional psychological attention.

### Participants

A total of 68,849 students in grades 7–12 completed our online survey within 3 weeks. In the present study, inclusion criteria for participation include: (a) age of 10–24 years; (b) no history of psychiatric diagnosis; and (c) response time of the online survey ≥5 min. Among the participants, 1,317 were excluded due to the failure to meet any of the aforementioned criteria. Finally, 67,532 students' data was qualified and included in the subsequent analyses.

### Measures

#### Sleep disturbance

Sleep disturbance in the past month was assessed by four items. Of the four items, based on criteria of the Diagnostic and Statistical Manual of Mental Disorders (38), three were used to measure insomnia symptoms, as follow: difficulty initiating sleep, difficulty maintaining sleep and waking up in early morning. Each item was rated on a 5-point scale, from 1 (never) to 5 (6–7 times/week). If any of the three items were rated as “≥3 times/week,” the participants were classified as having insomnia symptoms (39, 40). One additional self-reported item “During the past month, how would you rate the quality of your sleep overall?” was used to assess general sleep quality. Response to each item ranged from 1-very good, 2-good, 3-fair, 4-poor, to 5-very poor. A response of “poor” or “very poor” was considered as having poor sleep quality (41). Overall sleep disturbance was defined as having insomnia symptoms or poor sleep quality (42). The Cronbach's α was 0.81 of these items in this study.

#### PLEs

PLEs frequency over the past month was measured by the 8-item Positive Subscale of the Community Assessment of Psychic Experiences (CAPE-P8). The questionnaire was derived from the 42-item Community Assessment of Psychic Experiences (CAPE) (43). The scale was clustered into two dimensions: hallucinatory experiences (HEs, 6 items) and delusional experiences (DEs, 2 items). Each item could be answered from 1 (never) to 4 (nearly always), with higher total score indicating more occurrences of PLEs. The Chinese version of CAPE-P8 demonstrated satisfactory psychometric properties (44, 45) and was widely used in Chinese adolescent's study (4, 15). In this study, frequent PLEs were defined as having “often” or “nearly always” PLEs (4, 25). The Cronbach's α was 0.91.

#### Family function

Family function was assessed by family APGAR scale (46). It consists of 5 items: adaptation, partnership, growth, affection, and resolve. Each item was rated on a three-point Likert scale, from 0 (hardly ever) to 2 (almost always). Item scores were added up to generate a total score. Higher total score indicates better family function. The Cronbach's α was 0.93 in this study.

#### School climate

School climate was measured by the 2016 version of Delaware School Climate Scale-Student (DSCS-S) (47, 48). It was consisted of 31 items, which can be clustered into seven dimensions: teacher-student relations, student-student relation, fairness of rules, clarity of expectations, school safety, student engagement, bullying (reverse scoring). The respondent rated each item on a four-point scale, ranging from 1 (completely disagree) to 4 (completely agree). Item scores were added up to generate a total score. Higher total score indicates better school climate. The Cronbach's α was 0.96 in this study.

#### Socio-demographic

Socio-demographic characteristics in our study were collected, including sex, age, grade, ethnicity, single-child family, parental marital status, boarding status, parents' education, whether the parents have a stable job, life style (exercise, smoking, and alcohol intake), chronic physical illness, history of psychiatric diagnosis, and family history of mental disorders.

### Data analysis

In this study, the term LBC refers to the minors residing in their original domicile but has not lived with their parents for over 6 months, as either one or both parents have migrated (30). The χ^2^ tests (for categorical variables), and independent sample *t*-test or ANOVA *F*-test (for continuous variables) were applied to compare socio-demographic characteristic, sleep disturbance, PLEs and other variables between LBC and non-LBC. In order to examine the independent effect of sleep disturbance and parental migration on PLEs, a model of binary logistic regression analysis was conducted with overall sample. In all models of logistic regression analysis mentioned above, the dependent variable was PLEs (0 = none, 1 = frequent PLEs). Sleep disturbance (0 = no, 1 = yes) and parental migration (0 = no, 1 = yes) were computed as independent variables. We included family function, school climate, and socio-demographic characteristics (sex, age, ethnicity, single-child family, parental marital status, boarding status, parents' education, whether the parents have a stable job, exercise, smoking, and alcohol intake, chronic physical illness, and family history of mental disorders) as covariates. Furthermore, to explore the potential moderating effect of parental migration on the association between sleep disturbance and PLEs, a stratified analysis was also performed in LBC and non-LBC respectively. Additional logistic regressions were further conducted on the entire sample, using parental migration and the interaction terms for all variables suspected of having an effect on PLEs in both groups (LBC/non-LBC) as independent variables. If the interaction terms between these variables and parental migration are statistically significant, then it suggests that their effects on PLEs are different between LBC and non-LBC group. The results were displayed with odds ratios (ORs) and 95% confidence intervals (95% CIs). The statistically significance was defined as *p*-value of < 0.05. All statistical analyses were conducted using SPSS 23.0.

## Results

### Sample characteristics

We included 67,532 adolescents with 11, 821 LBC (17.5%) and 55,711 non-LBC (82.5%) in the final analysis. The proportion of male was slightly higher than female (53.7 vs. 46.3%). The participants ranged in age from 12 to 18 years, with the mean (SD) age was 15.18 (1.71) years. About three-quarters (74.9%) of the participants were junior high school students. About 19.9% were from single-child families and most of the students (96.3%) are of Han ethnicity. More detailed socio-demographic characteristics are presented in Table 1.

**Table 1:** Demographic characteristics of participants and differences between left-behind children (LBC) and non-LBC.

**Variables**		**Total** **(*n* = 67, 532)**	**LBC** **(*n* = 11, 821)**	**Non-LBC** **(*n* = 55, 711)**	**χ^2^/*t***	**Cramer's V/cohen's d**
Sex	Boys	36, 262 (53.7)	5, 968 (50.5)	30, 294 (54.4)	59.37^***^	0.030
	Girls	31, 270 (46.2)	5, 853 (49.5)	25, 417 (45.6)		
Age, years	M (SD)	14.56 (1.56)	14.71 (1.64)	14.53 (1.54)	10.84^***^	0.113
Grade (age)	7th [13.11 (0.83)]	20, 207 (29.9)	3, 232 (27.3)	16, 975 (30.5)	193.77^***^	0.054
	8th [14.01 (0.77)]	16, 691 (24.7)	2, 851 (24.1)	13, 840 (24.8)		
	9th [14.92 (0.76)]	13, 727 (20.3)	2, 214 (18.7)	11, 513 (20.7)		
	10th [15.79 (0.64)]	6, 922 (10.2)	1, 388 (11.7)	5, 534 (9.9)		
	11th [16.70 (0.62)]	6, 244 (9.2)	1, 290 (10.9)	4, 954 (8.9)		
	12th [17.71 (0.75)]	3, 741 (5.5)	846 (7.2)	2, 895 (5.2)		
Ethnicity	Han^a^	65, 038 (96.3)	11, 281 (95.4)	53, 757 (96.5)	30.85^***^	0.021
	Others	2, 494 (3.7)	540 (4.6)	1, 954 (3.5)		
Single-child family	Yes	13, 416 (19.9)	3, 117 (26.4)	10, 229 (18.5)	380.55^***^	0.075
	No	54, 116 (80.1)	8, 704 (73.6)	45, 412 (81.5)		
Parental marital status	Good	63, 455 (94.0)	9, 086 (76.9)	54, 369 (97.6)	7, 386.01^***^	0.331
	Poor^b^	4, 077 (6.0)	2, 735 (23.1)	1, 342 (2.4)		
Boarding options	School	19, 722 (29.2)	40, 761 (34.4)	15, 661 (28.1)	183.83^***^	0.52
	Home	47, 810 (70.8)	7, 760 (65.6)	40, 050 (71.9)		
Father' education	Primary school or less	3, 344 (5.0)	750 (6.3)	2, 594 (4.7)	73.30^***^	0.033
	Junior high	19, 511 (28.9)	3, 505 (29.7)	16, 006 (28.7)		
	Senior high school	20, 675 (30.6)	3, 584 (30.3)	17, 091 (30.7)		
	College or more	24, 002 (35.5)	3, 982 (33.7)	20, 020 (35.9)		
Mother' education	Primary school or less	6, 135 (9.1)	1, 165 (9.9)	4, 970 (8.9)	20.46^***^	0.017
	Junior high	21, 481 (31.8)	3, 845 (32.5)	17, 636 (31.7)		
	Senior high school	19, 163 (28.4)	3, 200 (27.1)	15, 963 (28.7)		
	College or more	20, 753 (30.7)	3, 611 (30.5)	17, 142 (30.8)		
Father has a stable job	Yes	62, 510 (92.6)	10, 489 (88.7)	52, 021 (93.4)	305.62^***^	0.067
	No	5, 022 (7.4)	1, 332 (11.3)	3, 690 (6.6)		
Mother has a stable job	Yes	53, 472 (79.2)	9, 451 (80.0)	44, 021 (79.0)	5.16^*^	0.009
	No	14, 060 (20.8)	2, 370 (20.0)	11, 690 (21.0)		
Exercise	Never	6, 129 (9.1)	1, 424 (12.0)	4, 705 (8.4)	239.28^***^	0.060
	Sometimes	26, 677 (39.5)	4, 950 (41.9)	21, 727 (39.0)		
	Often	34, 726 (51.4)	5, 447 (46.1)	29, 279 (52.6)		
Smoking	Never	67, 090 (99.3)	11, 675 (98.8)	55, 415 (99.5)	75.32^***^	0.033
	Sometimes	288 (0.4)	99 (0.8)	189 (0.3)		
	Often	154 (0.2)	47 (0.4)	107 (0.2)		
Alcohol intake	Never	63, 820 (94.5)	10, 800 (91.4)	53, 020 (95.2)	273.12^***^	0.064
	Sometimes	3, 579 (5.3)	980 (8.3)	2, 599 (4.7)		
	Often	133 (0.2)	41 (0.3)	92 (0.2)		
Chronic physical illness	Yes	2, 393 (3.5)	578 (4.9)	1, 815 (3.3)	75.97^***^	0.034
	No	65, 139 (96.5)	11, 243 (95.1)	53, 896 (96.7)		
Family history of mental disorders	Yes	593 (0.9)	196 (1.7)	397 (0.7)	100.15^***^	0.039
	No	66, 939 (99.1)	11, 625 (98.3)	55, 314 (99.3)		

^a^Han is the major ethnic group in China.

^b^Poor parental marital status included separated, divorced and widowed.

LBC, left-behind children.

^*^p < 0.05;

^***^p < 0.001.

The LBC group showed a significantly higher percentage of female participants (χ^2^ = 59.37, *p* < 0.001), older age (*t* = 10.84, *p* < 0.001) and a higher fraction of students enrolled in higher grades (χ^2^ = 193.77, *p* < 0.001). Other comparisons of two groups of characteristics were also displayed in Table 1.

### Prevalence of sleep disturbance and PLEs

Among the participants, 14.7% reported sleep disturbance and 15.5% reported frequent PLEs in the past month. As shown in Table 2, LBC showed higher prevalence of insomnia, poor sleep quality, overall sleep disturbance, HEs, DEs, and overall PLEs than non-LBC (all *p* < 0.001).

**Table 2:** The difference of sleep disturbance, PLEs and other variables between LBC and non-LBC.

**Variables**	**Total** **(*n* = 67, 532)**	**LBC** **(*n* = 11, 821)**	**Non-LBC** **(*n* = 55, 711)**	***p*-value**
**Sleep disturbance**, ***n*** **(%)**				
Insomnia	7, 893 (11.7)	1, 862 (15.8)	6, 031 (10.8)	< 0.001
Poor sleep quality	4, 534 (6.7)	1, 120 (9.5)	3, 414 (6.1)	< 0.001
Overall sleep disturbance	9, 950 (14.7)	2, 329 (19.7)	7, 621 (13.7)	< 0.001
**PLEs**, ***n*** **(%)**				
HEs	9, 627 (14.3)	2, 290 (19.4)	7, 337 (13.2)	< 0.001
DEs	3, 418 (5.1)	792 (6.7)	2, 626 (4.7)	< 0.001
Overall PLEs	10, 425 (15.4)	2, 447 (20.6)	7, 984 (14.3)	< 0.001

LBC, left-behind children; DEs, delusional experiences; HEs, hallucinatory experiences; PLEs, psychotic-like experience.

### Logistic regression analysis

Logistic regression results showed that sleep disturbance was associated with an increased risk of PLEs (OR = 4.04, 95% CI = 3.84–4.25) and parental migration was weakly associated with an increased odds of PLEs (OR = 1.19, 95% CI = 1.12–1.26), after adjusting for confounding variables. Meanwhile, family function, school climate were the protective factors of PLEs. Compared to the male group, female students had higher risk of PLEs (OR = 1.16, 95% CI = 1.10–1.06). Younger participants were less likely to report all PLEs. The following variables were still significantly associated with PLEs: boarding school, father with a stable job, exercise, alcohol intake, chronic physical illness, and family history of mental disorders (see Table 3).

**Table 3:** Multinomial logistic regression of PLEs among adolescents.

		**OR (95% CI)**	***p*-value**
Parental migration (ref. = no)		1.19 (1.12, 1.26)	< 0.001
Sleep disturbance (ref. = no)		4.04 (3.84, 4.25)	< 0.001
Family function score		0.86 (0.85, 0.86)	< 0.001
School climate score		0.98 (0.97, 0.99)	< 0.001
Girls (ref. = boys)		1.16 (1.10, 1.21)	< 0.001
Age, years		0.93 (0.91, 0.95)	< 0.001
Ethnicity Han (ref. = others)		0.94 (0.84, 1.06)	0.298
Single-child family (ref. = no)		1.08 (1.02, 1.15)	0.008
Poor parental marital status (ref. = good)		1.07 (0.97, 1.17)	0.179
Boarding school (ref. = home)		0.90 (0.85, 0.95)	< 0.001
Father's education (ref. = primary school or less)	Junior high school	0.94 (0.85, 1.05)	0.251
	Senior high school	0.93 (0.83, 1.04)	0.193
	College or more	1.02 (0.91, 1.14)	0.787
Mother's education (ref. = primary school or less)	Junior high school	0.97 (0.89, 1.06)	0.518
	Senior high school	1.02 (0.93, 1.120)	0.659
	College or more	1.04 (0.94, 1.15)	0.438
Father has a stable job (ref. = no)		0.86 (0.80, 0.93)	< 0.001
Mother has a stable job (ref. = no)		1.02 (0.97, 1.08)	0.483
Exercise (ref. = never)	Sometimes	0.93 (0.86, 1.00)	0.050
	Often	0.90 (0.83, 0.97)	0.008
Smoking (ref. = never)	Sometimes	1.17 (0.88, 1.56)	0.270
	Often	1.09 (0.74, 1.61)	0.672
Alcohol intake (ref. = never)	Sometimes	1.75 (1.60, 1.90)	< 0.001
	Often	1.98 (1.32, 2.96)	0.001
Chronic physical illness (ref. = no)		1.36 (1.23, 1.51)	< 0.001
Family history of mental disorders (ref. = no)		1.67 (1.38, 2.02)	< 0.001

PLEs, psychotic-like experience; OR, odds ratio; CI, confidence interval.

### Stratified analysis in LBC and non-LBC

In our study, sleep disturbance was strongly associated with PLEs with or without parental migration (see Table 4). LBC who reported sleep disturbance were at greater risk of having PLEs (OR = 3.84, 95% CI = 3.46–4.27). Similarly, non-LBC who reported sleep disturbance had increased odds of PLEs (OR = 4.09, 95% CI = 3.86–4.34). The interaction terms between sleep disturbance and parental migration are not statistically significant (OR = 0.94, 95%CI = 0.83–1.06), suggesting the assertion that the effect of sleep disturbance on PLEs is not different between the two groups holds. In addition, poor parental marital status significantly increase the risk of suffering frequent PLEs in the non-LBC group (OR = 1.21, 95%CI = 1.04–1.40), there is no such effect in the LBC group (OR = 0.99, 95%CI = 0.87–1.11), with the interaction term being statistically significant (*p* = 0.039).

**Table 4:** Multinomial logistic regression of PLEs in LBC and non-LBC.

		**LBC**		**Non-LBC**		**Interaction with parental migration**	
		**OR (95% CI)**	***p*** **-value**	**OR (95% CI)**	***p*** **-value**	**OR (95% CI)**	***p*** **-value**
Sleep disturbance (ref. = no)		3.84 (3.46, 4.27)	< 0.001	4.09 (3.86, 4.34)	< 0.001	0.94 (0.83, 1.06)	0.300
Family function score		0.85 (0.83, 0.86)	< 0.001	0.86 (0.85, 0.87)	< 0.001	0.99 (0.97, 1.01)	0.203
School climate score		0.98 (0.97, 0.99)	< 0.001	0.98 (0.97, 0.99)	< 0.001	1.00 (0.99, 1.00)	0.094
Girls (ref. = boys)		1.20 (1.10, 1.33)	< 0.001	1.14 (1.09, 1.20)	< 0.001	1.05 (0.94, 1.17)	0.411
Age, years		0.92 (0.89, 0.95)	< 0.001	0.93 (0.92, 0.95)	< 0.001	0.98 (0.95, 1.02)	0.428
Ethnicity Han (ref. = others)		1.01 (0.80, 1.33)	0.942	0.92 (0.80, 1.05)	0.207		
Single-child family (ref. = no)		1.15 (1.02, 1.29)	0.024	1.07 (1.00, 1.14)	0.064	1.06 (0.93, 1.21)	0.382
Poor parental marital status (ref. = good)		0.99 (0.87, 1.11)	0.440	1.21 (1.04, 1.40)	0.015	0.82 (0.67, 0.0.99)	0.039
Boarding school (ref. = home)		0.95 (0.85, 1.08)	0.440	0.88 (0.83, 0.94)	< 0.001	1.04 (0.87, 1.25)	0.316
Father's education (ref. = primary school or less)	Junior high school	0.87 (0.71, 1.08)	0.212	0.96 (0.85, 1.09)	0.539		
	Senior high school	0.83 (0.67, 1.03)	0.064	0.96 (0.84, 1.10)	0.554		
	College or more	0.94 (0.75, 1.18)	0.601	1.04 (0.91, 1.19)	0.547		
Mother's education (ref. = primary school or less)	Junior high school	1.11 (0.93, 1.33)	0.247	0.93 (0.85, 1.03)	0.160		
	Senior high school	1.05 (0.86, 1.27)	0.660	1.01 (0.81, 1.14)	0.863		
	College or more	1.11 (0.90, 1.36)	0.325	1.02 (0.91, 1.14)	0.741		
Father has a stable job (ref. = no)		0.89 (0.77, 1.04)	0.154	0.85 (0.77, 0.94)	0.001	1.04 (0.87, 1.25)	0.655
Mother has a stable job (ref. = no)		1.02 (0.90, 1.15)	0.782	1.02 (0.96, 1.09)	0.494		
Exercise (ref. = never)	Sometimes	0.95 (0.82, 1.11)	0.524	0.92 (0.84, 1.00)	0.054	1.04 (0.87, 1.24)	0.672
	Often	0.93 (0.80, 1.09)	0.364	0.89 (0.81, 0.97)	0.011	1.04 (0.87, 1.25)	0.639
Smoking (ref. = never)	Sometimes	1.17 (0.72, 1.90)	0.515	1.19 (0.84, 1.69)	0.330		
	Often	0.83 (0.42, 1.67)	0.608	1.27 (0.80, 2.03)	0.332		
Alcohol intake (ref. = never)	Sometimes	1.56 (1.33, 1.85)	< 0.001	1.82 (1.65, 2.02)	< 0.001	0.85 (0.70, 1.02)	0.085
	Often	1.37 (0.65, 2.88)	0.405	2.28 (1.41, 3.69)	0.001	0.56 (0.24, 1.33)	0.189
Chronic physical illness (ref. = no)		1.32 (1.08, 1.62)	0.007	1.38 (1.22, 1.56)	< 0.001	0.96 (0.76, 1.22)	0.747
Family history of mental disorders (ref. = no)		1.76 (1.27, 2.44)	0.001	1.62 (1.28, 2.06)	< 0.001	1.08 (0.72, 1.62)	0.697

LBC, left-behind children; PLEs, psychotic-like experience; OR, odds ratio; CI, confidence interval.

## Discussion

To our knowledge, this is the first study to examine the association between sleep disturbance and PLEs *via* a large sample of urban LBC and non-LBC in China. Our major findings are summarized as follow: (a) LBC showed significantly higher prevalence of sleep disturbance and PLEs than non-LBC in urban China; (b) Parental migration was a potential risk factor of PLEs after controlling for confounders; and (c) Sleep disturbance was a robust risk factor of PLEs, with or without the effect of parental migration. These findings provide some new insights on PLEs, which could benefit educators and psychologists to implement specific interventions on mental health problems among adolescents.

According to the definition of LBC (30), 17.5% of the students in our sample were classified as urban LBC. The percentage was much lower than those found in studies of rural or urban LBC. For instance, in a study of a representative sample of rural children (*N* = 15,232), Peng et al. (49) found that 24.6% of the children were left-behind. Another a small sample study (*N* = 796) in Chongqing, China, using random sampling, found that 41.7% of children were left behind in the urban (50). Potential explanations of the different percentage of LBC could be due to the differences in samplings and regions. In Shenzhen city, as families are generally better off financially, many parents also choose to bring their children with them and migrate to live together in their place of work.

In our study, both insomnia and poor sleep quality have higher prevalence in LBC, which was similar to one previous study in Western China (Sichuan Province) (32). Meanwhile, echoing with Sun et al.'s (25) study with mixed a sample of adolescents in rural and urban areas, this result showed no difference between LBC and non-LBC on frequent PLEs, while higher score of PLEs was found in LBC. Our data provide clearer evidence that urban LBC reported higher proportion of frequent PLEs than non-urban LBC. The direct implication of parental migration is parental absence. Evidence from studies in various countries supports that compared to children or adolescents living with parents, those from families where parents are absent tend to suffer more from emotional and behavioral symptoms, such as depression, anxiety, conduct disorders, and suicide behaviors (17). Our findings further indicated that parental migration was a potential risk factor of PLEs after controlling for some confounders, even if the effect was weak (OR = 1.19). This result is in line with a previous study, which demonstrated that LBC had significantly higher odds of PLEs with a weak effect size (OR = 1.19) (27). This can be disturbed for several reasons. On the one hand, LBC were more susceptible to lack of care and loneliness, resulting in higher risk of abuse and bullying (51), which in turn exacerbates the emergence of PLEs. In addition to the higher frequency of traumatic events observed among LBC, these children also reported more severe emotional impacts from these events compared to non-LBC, even after adjusting for the number of traumatic events, perhaps offering an explanation for the presence of more frequent PLEs in this group (25). On the other hand, compared to their non-LBC peers, LBC lack many protective factors against mental health. For instance, low self-concept (52) or lacking in resilience (53) can exacerbate the emergence of PLEs. These factors are all more likely to be identified among LBC (54, 55).

Our findings also indicate that sleep disturbance was a strong risk factor of PLEs after controlling for other covariates (OR = 4.04). The finding is in line with most previous studies (15, 56, 57), showing that participants with sleep disturbance generally have higher rates of PLEs. A range of sleep disturbance symptoms can be common presentation in adolescents with psychosis (58, 59). These sleep disturbance related symptoms are known to impair stress tolerance, immunological functioning, and cognitive functioning, as well as to exacerbate socioemotional distress (11, 12), all of which are considered to involve in the etiology of psychosis (13, 14). Evidence shows that neurological structures responsible for sleep regulation (e.g., thalamus, cortical gray matter) can also be affected in individuals who are further along the psychosis continuum (60). Moreover, based on our results, sleep disturbance increases the occurrence of PLEs among adolescents regardless the history of parental migration, which suggests a relatively stable effect of sleep disturbance on PLEs. Irrespective of their LBC or non-LBC status, adolescents are at higher risk of PLEs if they suffer from sleep disturbance. Numerous studies suggested that change of family structure could constitute a great source of stress among LBC by strengthening the impact of adversity on them (61). However, we argue that compared to LBC living in rural areas, the negative impact of parental migration may be less severe for urban LBC. There are some possible reasons to explain this observation. First, as transportation between urban areas is more developed, parents have more opportunities to visit their children on rest days, which buffer the impact of separation. Second, due to the educational resource discrepancy between urban and rural areas, urban LBC are also likely to receive more support from teachers and school psychologists. Collectively, despite the overall negative psychological impact of parental migration, there may be nuances between urban-to-urban and rural-to-urban ones. Further research is needed to elaborate the specific differences between these two groups of LBC.

Furthermore, environmental factors were found to associate with PLEs in adolescents regardless of parental migration. Our findings suggest that poor family function is a well-established risk factor for PLEs in the present sample, which is same with previous studies. For example, Adewuya et al. (62) has established a significant association between physical punishments at home and PLEs with an odd ratio of 1.98. Crush et al. (63) also indicated that the positive effect of low level of family support on the occurrence of PLEs. We have also identified a negative association between school climate and PLEs, which also consolidates the findings in a study by Liu et al. (64), who demonstrated that poor teacher-student relationship negatively predicted poor psychological outcomes. Therefore, improving both family function and school climate is crucial to protect the mental health of LBC and non-LBC. Moreover, consistent with previous studies (26, 65, 66), we also found that several risk factors such as being female, younger age, alcohol intake, chronic physical illness, and family history of mental disorders were associated with PLEs in adjusted analyses. In addition, our finding suggested that poor parental marital status is a weak predictor of frequent PLEs among non-LBC, while no association was found in the group of LBC. This may be due to the fact that the left-behind status causes the child to be unclear about their parents' marital status. Collectively, these factors should also be taken into consideration for implementing psychosocial interventions among students.

Some limitations for the study should be considered. First, sleep disturbance and PLEs were assessed by self-report rather than clinical diagnostics in the present study, as well as definition of sleep disturbance (4 items) differs from the commonly used sleep questionnaires (e.g., Pittsburgh Sleep Quality Index, Insomnia Severity Index), which may cause augment reporting, recall bias or underestimation of some psychological symptoms. Second, the cross-sectional nature of this study can only capture the correlational association between sleep disturbance and PLEs. A longitudinal design is required to clarify this result in the future research. Third, all of the participants are from Shenzhen city, despite the adequate sample size. It is uncertain whether our findings can be generalized to students in other parts of the country. Finally, several potential confounding factors were neglected in the analysis, such as the length of parental migration, the type of parental migration (e.g., single or both parents' absence), and the frequency of communication between parents. These factors need to be discussed in more detail in future research.

## Conclusion

Regardless the status of parental migration, sleep disturbance is positively associated with PLEs among Chinese urban adolescents. Our findings extend prior literature by first exploring the association between sleep disturbance and PLEs in LBC and non-LBC. In practice, adolescents' PLEs preventive strategies should focus on reducing sleep disturbance related symptoms, alcohol intake as well as improving family function and school climate, especially among higher risk individuals with the characteristics of being younger, female, having chronic physical illness or family history of mental disorders.

## Data availability statement

The original contributions presented in the study are included in the article/supplementary material, further inquiries can be directed to the corresponding author.

## Ethics statement

The studies involving human participants were reviewed and approved by the Ethics Board of the South China Normal University. Written informed consent to participate in this study was provided by the participants' legal guardian/next of kin.

## Author contributions

DW and FF: conceptualization. DW and ZM: data curation, methodology, formal analysis, and writing—original draft. SZ and MS: writing—review and editing. FF: resources, writing—review and editing. All authors contributed to the article and approved the submitted version.
